# The impact of the COVID-19 Pandemic on rhegmatogenous retinal detachment treatment patterns

**DOI:** 10.1186/s12886-021-02127-7

**Published:** 2021-10-19

**Authors:** Jipeng Li, Meng Zhao, Haicheng She

**Affiliations:** Ophthalmology, Beijing Tongren Eye Center, Beijing Key Laboratory of Ophthalmology and Visual Science, Beijing Tongren Hospital, Capital Medical University, No1. Dongjiaominxiang street, Dongcheng District, Beijing, 100730 China

**Keywords:** COVID-19 pandemic, Rhegmatogenous retinal detachment, Surgery

## Abstract

**Backgrounds:**

To describe changes in rhegmatogenous retinal detachment (RRD) surgical procedures in Beijing during the COVID-19 Pandemic.

**Methods:**

A retrospective cohort of RRD patients was analyzed. Patients were divided into the COVID-19 pandemic group and pre-COVID-19 group according to their presentation. The presurgery characteristics, surgical procedures, and surgery outcomes were collected. The potential factors related to the choice of pars plana vitrectomy (PPV) or scleral buckling (SB) were analyzed using logistic regression. The differences in the procedure choice under specific conditions were compared. Surgery outcomes were compared between the two groups.

**Results:**

In the COVID-19 pandemic group, less patients received SB (27.8, 41.3%, *p* = 0.02) while more patients received PPV (72.2, 58.6%, *p* = 0.02); in patients who received SB, fewer patients received subretinal fluid drainage (45.4,75.7%, *p* = 0.01); in patients who received PPV, fewer patients received phacovitrectomy (7.0, 21.0%, *p* = 0.02). The choice of PPV was related to older age (1.03, *p* = 0.005), the presence of RRD with choroidal detachment (RRD-CD) (2.92, *p* = 0.03), pseudophakia (5.0, *p* = 0.002), retinal breaks located posterior to the equator (4.87, *p* < 0.001), macular holes (9.76, *p* = 0.005), and a presurgery visual acuity (VA) less than 0.02 (0.44, *p* = 0.03). Fewer phakia patients with retinal breaks located posterior to the equator (1/28, 11/30, *p* = 0.01) and fewer patients with chronic RRD and subretinal strand (1/9, 9/16, *p* = 0.03) received SB in the COVID-19 pandemic group. There were more patients with improved VA (55.7, 40.2%, *p* = 0.03) in the COVID-19 pandemic group. The overall single-surgery retinal attachment rate was similar in the two groups (94.9, 94.5%, *p* = 0.99).

**Conclusions:**

During the COVID-19 Pandemic, the main reason for the increased number of PPV in RRD treatment was that more complicated cases were presented. However, the surgeons were conservative in procedure choice in specific cases. The adjustments on RRD treatments lead to comparable surgery outcomes.

## Background

Rhegmatogenous retinal detachment (RRD) is a vision-threatening emergency condition characterized by separating the neurosensory retina from the underlying retinal pigment epithelium, requiring emergent treatment [1, 2]. The current repairment procedures for RRD include pneumatic retinopexy, pars plana vitrectomy (PPV), and scleral buckling (SB) [3]. The procedure chosen depends on patients’ preoperative characteristics and surgeons’ preferences [4]. Previous reports focusing on RRD progression found that prolonged preoperative waiting time is related to the development of macular-off RRD [5], irreversible macular damage [6–8], proliferative vitreoretinopathy (PVR) progression [9], and development of choroidal detachment (CD) [10]. In addition, previous studies have shown that during national meetings or holiday-time, reduced staffing is often associated with adverse patient outcomes, changes in treatment patterns, and care delays in acute cardiovascular conditions [1, 11, 12] and RRD patients [13].

It is known that the front-line healthcare workers are at increased risk for the COVID-19 infection [14]. In particular, ophthalmologists are one of the three subspecialties with the highest risk of COVID-19 infection since the eye surface and the nasopharyngeal mucosa, expressing angiotensin-converting enzyme two (ACE2) receptors, are the exposed surfaces amenable to contagion [15, 16]. Since March 11, 2020, widespread changes and restrictions to social and sanitary practices have created significant eye-care access issues during the COVID-19 Pandemic [17]. Additionally, several national ophthalmology societies recommended reducing outpatient visits and postponing any treatment other than urgent or emergent care to contain human to human viral transmission and diverging resources to intensive care units, thus experiencing a dramatic drop in the care providing. Several authors have already discussed the ethical impact of delivering treatments to patients in need since the beginning of the COVID-19 outbreak [18]. For example, the EUROCOVAT group has reported changes in elective cataract surgeries [19], intravitreal injections [20], and corneal donors [21].

Furthermore, a framework has been reported in patients receiving intravitreal injections suffering from sight-threatening conditions when the available resources are limited, precluding all patients’ accommodation [19, 22]. A recent study from the US noted a decline in SB procedures and an increase in PPV on RRD patients in the non-COVID-19 period [23]. RRD surgical repairment was one of the remaining few ophthalmological surgery services during the COVID-19 Pandemic in Beijing. Here, all admitted patients needed a 14-day self-quarantine and negative COVID-19 test before they were accepted, and RRD surgeries were performed with limited operation resources, restricted general anesthesia, and limited follow-up visits. The research of COVID-19’s impact has tended to focus on the RRD characteristics [24, 25] rather than the procedures the surgeons chose. Accordingly, the impact of the COVID-19 Pandemic on RRD treatment is still unknown. This study, therefore, set out to assess the influence of COVID-19 on RRD procedure choice in a consecutive cohort of hospitalized RRD patients from the pre-COVID-19 period and during the COVID-19 Pandemic.

## Methods

This is a retrospective cohort study of surgeries from October 8, 2019, to April 30, 2020. We identified all hospitalized patients with RRD who underwent surgery at Beijing Tongren Eye Center during this period and divided them into the pre-COVID-19 pandemic group and the COVID-19 pandemic group, depending on when they were admitted for surgery. Calendar dates from February 10 to April 30, 2020, were used for the COVID-19 Pandemic period (the COVID-19 pandemic group) when the top-level response was carried out in Beijing. Calendar dates from October 8 to December 30, 2019, were used for the pre-COVID-19 period (the pre-COVID-19 group). The charts of enrolled patients were reviewed. This study was approved by the Ethics Committee of Beijing Tongren Hospital and adhered to the Declaration of Helsinki’s tenets. The informed consent was waived. The work has been reported in line with the STROCSS criteria [26].

The patient presurgery characteristics were collected, including presurgery wait time, the previous history of eye trauma, age, gender, PPV or SB, presurgery VA, retinal break location, lens status, the combination of pathological myopia (PM), the extent of retinal detachment, concomitant PVR, and CD. We also collected data on the specific surgical procedures. The outcome of VA and single surgery retinal attachment rate (SSRA) at 3 months after the surgery were investigated.

### Statistical analysis

Statistical analysis was performed using version 3.20 of R (http://www.R-project.org). Patient characteristics were retrieved from their medical charts and recorded in version 2.0.3.15 of EpiData Entry Client (http://epidata.dk). VA results were converted to logMAR values for statistical analysis. VA improvement was defined as a final VA gain of more than two lines. Mean and standard deviation (SD) values were calculated for continuous variables with a normal distribution. Medians with quartile values were calculated for continuous variables with a non-normal distribution. The t-test or Mann-Whitney U test was carried out for continuous variables. The chi-square test or Fisher’s exact test were carried out for discrete data.

To investigate the factors related to PPV or SB choice, we divided the patients into the PPV group and the SB group, depending on the procedure the patient received. The presurgery patients’ characteristics were compared between the two groups. Factors with *p* < 0.1 were enrolled in a binary backward stepwise logistic regression model. One variable was included or excluded from the model each time by comparing the Akaike information criterion (AIC) value, and the model with the lowest AIC was chosen. The Receiver Operator Characteristic (ROC) curve was plotted, and the area under the curve (AUC) was calculated for each logistic regression model.

To investigate the surgeons’ choice differences in the presence of specific potential risk factors in the two different periods, we divided the patients into the COVID-19 pandemic group and pre-COVID-19 group, depending on the patients’ presentation. The ratio of PPV/SB was calculated and compared between groups.

The VA improvement was defined as the final VA increased three lines or more from the baseline VA. The percentage of patients with VA improvement and SSRA were compared between the two groups with chi-square test or Fisher’s exact test.

## Results

### Changes in the treatment patterns (Table 1)

We identified 258 inpatients with RRD in our study. Among these patients, 79 were admitted during the COVID-19 Pandemic, and 179 were admitted during the pre-COVID-19 period.

**Table 1:** The difference in treatment patterns between the two groups

(n, %)	The COVID-19 pandemic group (79)	The pre-COVID-19 group (179)	P
PPV (n, %)	57, 72.2%	105, 58.6%	0.04
combined with SB	1, 1.8%	0, 0%	0.31
combined with PHACO	4, 7.0%	22, 21.0%	0.02
silicone oil tamponade	52, 91.2%	91, 86.7%	0.45
C3F8 tamponade	5, 8.8%	14, 13.3%	0.79
SB (n, %)	22, 27.8%	74, 41.3%	0.01
segmental buckle	8, 25.8%	15, 11.5%	0.06
radial buckle	1, 3.2%	6, 4.6%	0.59
combined encircling	13, 59.1%	53, 71.6%	0.62
drainage of subretinal fluid	10, 45.4%	56, 75.7%	0.01

Compared to the pre-COVID-19 group, fewer patients received SB (27.8, 41.3%) while more patients received PPV (72.2, 58.6%, *p* = 0.02). In patients who received SB, fewer patients received subretinal fluid drainage (45.4, 75.7%, *p* = 0.01) in the COVID-19 pandemic group. In patients who received PPV, fewer patients received PPV combined with cataract extraction (7.0, 21.0%, *p* = 0.02) in the COVID-19 pandemic group. The prevalence of silicone oil (91.2, 86.7%) or gas (8.8, 13.3%) tamponade in patients was similar between the two groups (*p* = 0.45).

### The impact of patients’ presurgery characteristics on the treatment patterns

The patients in the COVID-19 pandemic group had longer median presurgery waiting times than patients in the pre-COVID-19 group (28 days, 4 days, *p* < 0.001). The rate of RRD-CD (34.2, 19.6%, *p* = 0.01), pseudophakia (22.8, 13.4%, *p* = 0.047), and retinal breaks located posterior to the equator (48.1, 22.9%, *p* < 0.001) were higher in the COVID-19 pandemic group than in the pre-COVID-19 group.

The presurgery characteristics of the PPV group and SB group were listed in Table 2. There was a significant difference in age (*p* < 0.001), prevalence of RRD-CD (*p* < 0.001), recurrent RRD (*p* = 0.04), VA less than 0.02 (*p* < 0.001), VA of 0.1–0.5 (*p* < 0.001), VA greater than 0.5 (*p* = 0.01), pseudophakia (*p* = 0.004), location of retinal breaks (*p* < 0.001), macula-off status (*p* < 0.001), and PVR B-C (*p* < 0.001) during the COVID-19 pandemic (*p* = 0.054) between the two groups (Table 2).

**Table 2:** The patient presurgery characteristics in PPV and SB group

	PPV (162)	SB (96)	*P*
group (COVID/pre-COVID)	57/105	22/74	0.054
gender (male/female)	100/62	60/36	1
age (mean±SD, y)	52.7 + 13.6	39.9 + 19.9	< 0.001
presurgery waiting time (median, d)	4 [2,8]	4 [3,12]	0.51
RRD-CD (n)	55	17	< 0.001
dense VH (n)	7	3	0.74
recurrent RRD (n)	19	4	0.04
VA less than 0.02 (n)	99	24	< 0.001
VA 0.02-0.1 (n)	45	31	0.48
VA 0.1-0.5 (n)	13	30	< 0.001
VA greater than 0.5 (n)	5	11	0.01
PM (n)	57	23	0.08
pseudophakia (n)	35	7	0.004
retinal breaks location (n)			
anterior	69	82	< 0.001
posterior	67	12	< 0.001
macular hole (n)	2	26	0.0003
macular-off (n)	15	33	< 0.001
PVR B-C (n)	128	96	< 0.001

**Fig. 1:**
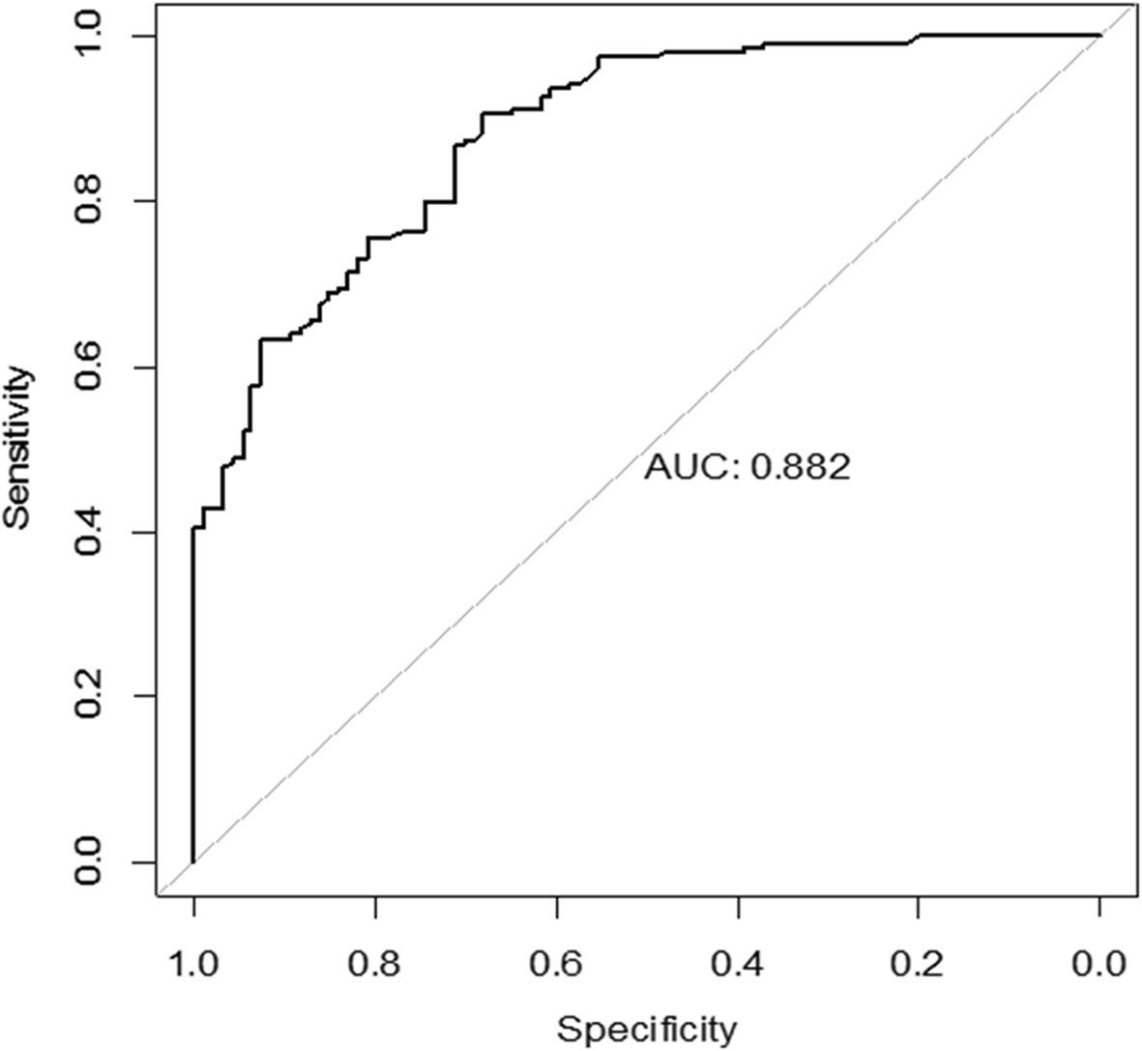
The ROC curve of the logistic regression model for factors related to the choice of PPV

Factors related to the choice of PPV in the logistic regression model were older, with the presence of RRD-CD and pseudophakia, retinal breaks located posterior to the equator, a macular hole, and presurgery VA less than 0.02 (AIC = 226.14, AUC = 0.882) (Fig. 1 and Table 3). Those presenting severe PVR, recurrent RRD, or PM during the COVID-19 Pandemic were excluded from the model.

**Table 3:** The factors related to the choice of PPV in the logistic regression model

	OR	CI	*P*
RRD-CD	2.92	1.09-7.89	0.03
psedophakia	5.00	1.81-14.08	0.002
retinal breaks posterior to equator	4.87	2.11-11.20	< 0.001
macular hole	9.76	2.01-47.41	0.005
age	1.03	1.01-1.05	0.005
VA less than 0.02	0.44	0.21-0.95	0.03

**Fig. 2:**
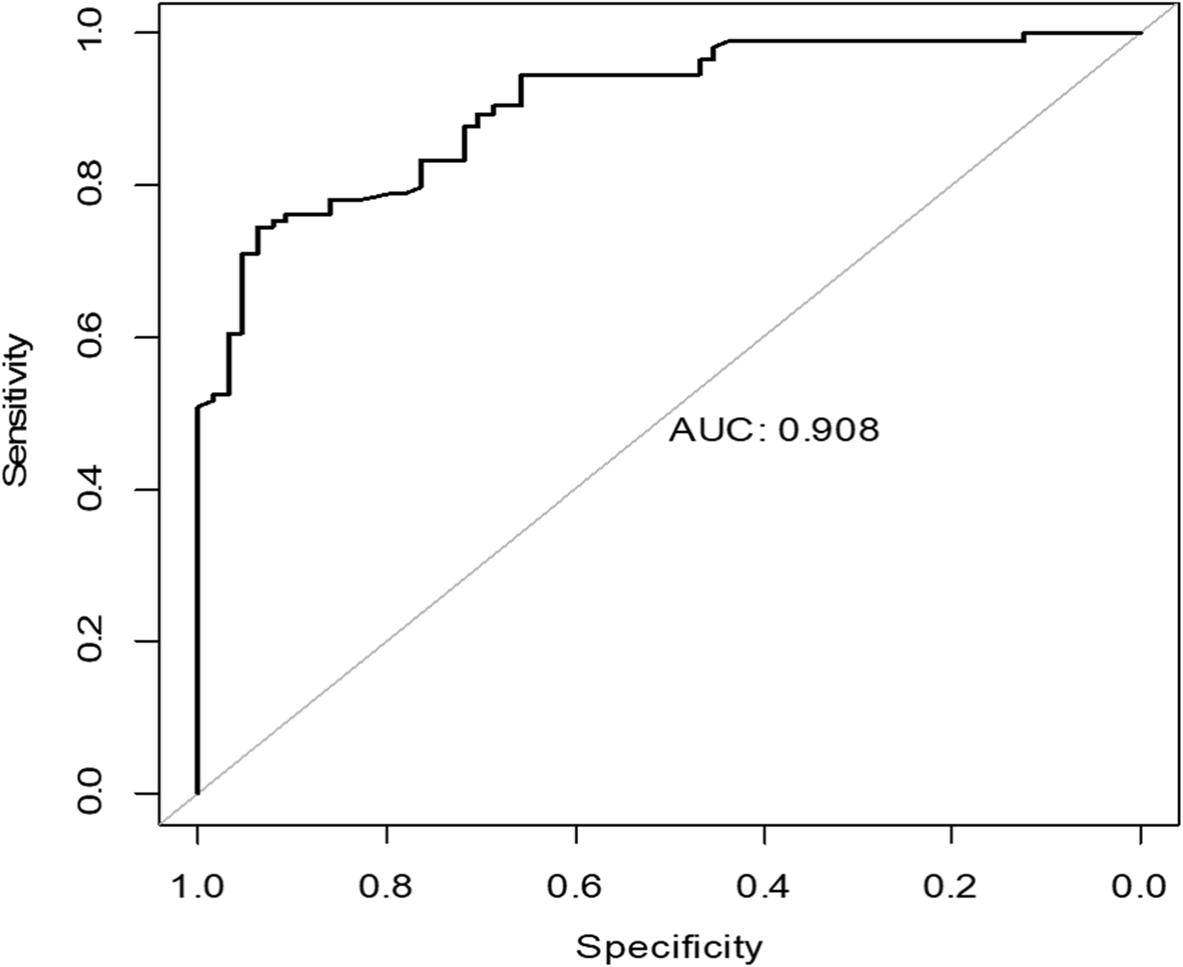
The ROC curve of the logistic regression model for factors related to the choice of SB

Factors related to the choice of SB in the logistic regression model were younger age, with the absence of RRD-CD and phakia, retinal breaks located anterior to the equator, and presurgery VA less than 0.02 (AIC = 249.69, AUC = 0.846) (Fig. 2 and Table 4). Those presenting severe PVR, recurrent RRD, or PM during the COVID-19 Pandemic were excluded from the model.

**Table 4:** The presurgery characteristics related to the difference of PPV/SB ratio in the two groups

	The COVID-19 pandemic group	The pre-COVID-19 group	*p*
age < =40	11/11	18/42	0.08
age > 40	46/11	87/32	0.18
RRD-CD	25/2	30/5	0.46
retinal breaks anterior to the equator	17/20	52/62	0.59
retinal breaks posterior to the equator	37/1	30/11	0.002
macular hole	4/0	23/1	0.85
pseudophakia	17/1	18/6	0.10
VA < 0.02	43/5	56/19	0.03

### The impact of the surgeon’s decision changes on the treatment patterns

We further investigated the difference of procedure choice between the two groups in the presence of specific factors mentioned above that favor PPV or SB. We compared the ratio of PPV/SB between the two groups on each factor enrolled in the logistic regression model (Table 4). We showed that the PPV/SB ratio was similar between the two groups in age (*p* = 0.18), RRD-CD(*p* = 0.46), retinal breaks located anterior to the equator (*p* = 0.59), macular hole (*p* = 0.85), and pseudophakia (*p* = 0.1). The PPV/SB ratio was different between the two groups in patients with retinal breaks located posterior to the equator (*p* = 0.002) and VA less than 0.02 (*p* = 0.03).

We showed fewer surgeons selected SB in patients with retinal breaks located posterior to the equator in the COVID-19 Pandemic (1,11, *p* = 0.002). In those patients, there were more pseudophakic patients in the COVID-19 pandemic group than the pre-COVID-19 group (9/38, 0/41, *p* < 0.001). There was no significant difference between the two groups in age (52.2, 51.9, *p* = 0.66), presurgery waiting times (5.1, 7.1, *p* = 0.16), severe PVR (32/38, 28/41, *p* = 0.11), RRD-CD (21/38, 30/41, *p* = 0.11), PM (11/38, 7/41, *p* = 0.28), or macula-off (35/38, 34/41, *p* = 0.31).

Aside from the impact of pseudophakia on the treatment pattern, there were fewer phakia patients with retinal breaks located posterior to the equator who received SB in the COVID-19 pandemic group than in the pre-COVID-19 group (1/28, 11/30, *p* = 0.01). There were seven patients in the pre-COVID-19 group, and none in the COVID-19 pandemic group received radial buckling.

We also showed fewer patients whose VA was less than 0.02 received SB in the COVID-19 pandemic group than in the pre-COVID-19 group (5/48, 19/75, *p* = 0.03). Those patients whose VA was less than 0.02 had a higher prevalence of retinal breaks located posterior to the equator (31/48, 17/75, *p* < 0.001), and there were shorter presurgery waiting times (4.8, 17.0 months, *p* = 0.008) in the COVID-19 pandemic group than in the pre-COVID-19 group. There was no significant difference between the two groups in age (54.0, 51.4 years, *p* = 0.36), combination of PM (14/48, 27/75, *p* = 0.56), RRD-CD (22/48, 23/75, *p* = 0.13), pseudophakia (11/48, 8/75, *p* = 0.08), severe of PVR (41/48, 56/75, *p* = 0.23), or macular-off status (41/48, 71/75, *p* = 1.0). Most patients with retinal breaks located posterior to the equator in the two groups received PPV (31/31,15/17, *p* = 0.12). The patients with retinal breaks located anterior to the equator presented with chronic RRD patients with PVR C; fewer of them received SB in the COVID-19 pandemic group than in the pre-COVID-19 group (1/9, 9/16, *p* = 0.03).

### The impact of the surgeon’s decision changes on the adjunctive procedures

We showed fewer patients who received cataract extraction combined with PPV in the COVID-19 pandemic groups (4/57, 22/105, *p* = 0.02). There was no significant difference in patients’ presurgery characteristics for those who received combined surgery or those who received PPV in the two groups (Table 5).

**Table 5:** The presurgery characteristic of patients in the two group underwent combined surgery or PPV

	Patients underwent combined surgery			Patients underwent PPV		
	The COVID-19 pandemic group(52)	The pre-COVID-19 group(83)	*p*	The COVID-19 pandemic group(4)	The pre-COVID-19 group(22)	*p*
age (mean, y)	53.7	52.3	0.51	44.5	52.1	0.32
presurgery wait times (median, d)	5.0	6.2	0.18	6.5	43.6	0.01
RRD-CD (n)	22	22	0.09	3	8	0.27
PM (n)	16	32	0.46	1	8	1.0
PVR B-C (n)	47	65	0.10	1	14	0.27
macular hole (n)	1	13	0.02	2	10	1.0
retinal breaks anterior to the equator (n)	15	46	0.004	1	6	1.0
retinal breaks posterior to the equator (n)	36	24	0.001	1	6	1.0

We showed fewer patients who received subretinal fluid drainage in SB surgery (10/22, 56/74, *p* = 0.01). In those without subretinal fluid drainage, the rate of PM in the COVID-19 pandemic group was higher than in the pre-COVID-19 group (9/13, 5/28, *p* = 0.003), and the presurgery waiting times were shorter in the COVID-19 pandemic group than in the pre-COVID-19 group (4.2, 15.3 months, *p* = 0.01). There was no significant difference in other presurgery characteristics between the two groups. There was no significant difference in presurgery characteristics in patients who received subretinal fluid drainage between the two groups (Table 6).

**Table 6:** The presurgery characteristic of patients in the two group underwent subretinal fluid drainage or without subretinal fluid drainage

	Patients without subretinal fluid drainage			Patients underwent subretinal fluid drainage		
	The COVID-19 pandemic group(13)	The pre-COVID-19 group(28)	*p*	The COVID-19 pandemic group(11)	The pre-COVID-19 group(46)	*p*
age (mean, y)	32	35.3	0.62	48.2	42.4	0.36
presurgery wait time (median, d)	4.2	15.3	0.01	11.3	7.1	0.33
RRD-CD (n)	1	0	0.31	2	5	0.61
PM (n)	9	5	0.003	2	6	0.64
pseudophakia (n)	1	3	1.0	0	3	1.0
PVR B-C (n)	9	20	0.40	9	38	1.0
retinal breaks posterior to the equator (n)	2	3	0.65	0	8	0.33
retinal breaks anterior to the equator (n)	11	25	0.65	11	37	0.18
macular-off (n)	10	14	0.17	9	31	0.48

### The outcomes of surgery

Compared to patients in the pre-COVID-19 group, the percentage of patients with VA improvement was higher in the COVID-19 group (55.7, 40.2%, *p* = 0.03).

The overall SSRA rate was similar in the two groups (94.9, 94.5%, *p* = 0.99). Also, the SSRA rate of PPV (96.5, 94.3%, *p* = 0.80) and SB (90.9, 94.6%, *p* = 0.61) was similar in the two groups.

One out of seven patients with a retinal break located posterior to the equator who received radial buckling and one out of two pseudophakic patients with macular on status in the pre-COVID-19 group failed the SB surgery and required secondary surgery.

## Discussion

The COVID-19 Pandemic significantly impacted clinical work. Surgeons perform retinal detachment repairment in the most challenging conditions when facing the reduction of outpatient service and operative resources, the restricted use of general anesthesia, limited follow-up visits, and more complicated cases. This study investigated the real-world data on the changes in RRD treatments in the COVID-19 Pandemic. We found that the surgeons’ choice of the procedure depended mainly on the patients’ characteristics and was not influenced by the COVID-19 Pandemic. However, when facing more complicated cases, the surgeons were more conservative, choosing PPV instead of SB and giving up subretinal fluid drainage in SB cases or cataract extraction in PPV cases. Fortunately, in the COVID-19 pandemic period, the treatment changes in more complicated cases led to a VA and retinal attachment rate that was comparable to the same rate in the pre-COVID-19 period.

To rule out the impact of long holidays on the treatment changes, we did not include patients who presented during the Chinese Spring Festival. To rule out the impact of surgeon preference and reshaping of retinal residents training during COVID-19 [27] on the treatment, we selected a cohort of patients treated by the same group of professor surgeons.

It has been reported that pseudophakia, older age, location and number of retinal breaks, and vitreous hemorrhage are related to PPV choice, while lattice degeneration and younger age are associated with SB choice [4, 23, 28]. Other factors known to contribute to the selection of PPV over SB are macular holes, giant tears, severe PVR [29], and RRD-CD [30, 31]. Similar to the previous works, we found that pseudophakia, RRD-CD, older age, retinal breaks located posterior to the equator, macular holes, and worse VA were associated with the selection of PPV over SB.

We found that the prevalence of RRD-CD, pseudophakia, retinal breaks located posterior to the equator, and VA less than 0.02 was higher in the COVID-19 pandemic group than in the pre-COVID-19 group. The more complicated cases presented during the COVID-19 Pandemic may contribute to the increased number of patients who received PPV.

Meta-analysis notes that the retinal attachment rate in pseudophakic eyes performed with PPV is higher than with SB [3]. The recurrence of RD occurs more frequently in pseudophakic eyes due to PVD, vitreous base traction, and multiple undetected breaks [32]. We found that pseudophakia prevalence was 22.8% in the COVID-19 Pandemic, higher than 13.4% in the pre-COVID-19 period, which may contribute to the increased PPV performed during the COVID-19 Pandemic.

The prevalence of RRD-CD is 8.6–19.62% [10, 33].RRD-CD is related to the progression of PVR [34] and recurrent RRD [35]. The retinal attachment rate for PPV in RRD-CD patients is reported as 72.57% [36]. RRD-CD prevalence was 34.2% in the COVID-19 Pandemic, higher than 19.6% in the pre-COVID-19 period. It may also contribute to the increased PPV performed during the COVID-19 Pandemic.

The vitreoretinal traction is related to developing the retinal break located posterior to the equator and RRD [4, 37]. In SB surgery, for patients with retinal tears posterior to the equator, releasing the vitreoretinal traction requires a careful buckle selection and orientation tailored to the tear [8, 9, 15]. The inadequate buckle may lead to the opening of the retinal break and surgery failure [38]. On the contrary, PPV has an advantage in dealing with the vitreoretinal traction and closing the break at the same time [3, 39]. The higher prevalence of retinal break located posterior or to the equator was found in the COVID-19 pandemic group (48.1%), which may contribute to PPV selection.

Altogether, the selection of PPV over SB in our study was mainly based on the presurgery RRD characteristics. The increased number of complicated cases may result in the increased PPV in the COVID-19 Pandemic.

We then investigated the difference in treatment patterns between the COVID-19 pandemic group and the pre-COVID-19 group in patients with certain similar conditions.

We found fewer patients with retinal break located posterior to the equator who received SB in the COVID-19 pandemic group. Since there were more pseudophakia patients in the COVID-19 pandemic group, we investigated the treatment pattern in phakia patients. Even in phakia patients, fewer patients in the COVID-19 pandemic group received SB than the pre-COVID-19 group. In patients with retinal break located posterior to the equator who received SB, seven patients in the pre-COVID-19 group and none in the COVID-19 pandemic group received radial scleral buckling. Several considerations can explain the reluctance of performing SB in patients with retinal break posterior to the equator in the COVID-19 Pandemic: 1) there was restricted use of general anesthesia in the COVID-19 Pandemic, and patients had inadequate compliance under local anesthesia and could not tolerate the complicated SB surgery; 2) the SB procedure for patients with retinal break located posterior to the equator is time-consuming, and it was challenging for the surgeon to perform the complicated SB surgery under enforced medical protective equipment used against COVID-19 virus contamination; 3) it was challenging to arrange intense follow-up visits and secondary surgery during the COVID-19 Pandemic.

We found fewer patients in the COVID-19 pandemic group received combined phacovitrectomy than the pre-COVID group when the patients in both groups had similar presurgery RRD characteristics. Besides the longer surgery time of the combined procedure, the combined phacovitrectomy may lead to elevated IOP, anterior chamber fibrin reaction, posterior synechia [40, 41], and more significant postoperative refractive prediction error [42]. It is challenging to arrange frequent follow-up to monitor the anterior chamber reaction and IOP in the COVID-19 Pandemic. The shortage of biometry examination for intraocular lens calculation made the surgeons postpone the combined cataract extraction surgery due to the disinfection process.

Subretinal fluid drainage is commonly performed during SB [38, 43] and may cause subretinal hemorrhage, retinal perforation, drainage of the liquefied vitreous, vitreoretinal incarceration, eye hypotony, and choroidal detachment [44]. However, only a few cases of subretinal fluid drainage were noted during the COVID-19 Pandemic. The risk of subretinal fluid drainage complications and the difficulties of treating SB’s complications are the main concerns in performing SB during COVID-19. Therefore, a drop in the number of subretinal fluid drainage cases was noted during the COVID-19 Pandemic.

We further investigated the RRD characteristics in patients who received SB alone. More PM patients received SB alone during the COVID-19 Pandemic. PM is known as the risk factor for developing CD after scleral buckling [45]. The fear of subretinal fluid drainage complications, mostly when patients are under local anesthesia, makes the surgeons give up the adjunctive procedure or turn to PPV instead.

Regarding patients with chronic RRD and retinal breaks located anterior to the equator, one patient in the COVID-19 group and nine patients in the pre-COVID-19 group received SB. SB provides an advantage in dealing with chronic RRD with a subretinal brand and atrophic breaks [43], but 11.3% of patients were reported to undergo the secondary procedure to deal with an unattached retina [46]. PPV is reserved for complicated cases and is often accompanied by retinoctomy and silicone oil tamponade to achieve better outcomes [47, 48]. The requirement of multiple follow-ups concerning the persistence of a detached retina, a fear of PVR progression [49] when the break is located near the subretinal brand, and bad toleration of the SB procedure under local anesthesia may partly account for the changes of treatment towards PPV.

In conclusion, due to general anesthesia restrictions and limitations on clinics and operative resources during the COVID-19 Pandemic, surgeons were more conservative with certain kinds of RRD cases.

The treatment pattern was changed by the presurgery characteristics and surgeons’ considerations during the COVID-19 Pandemic. Contrary to the previous report of higher prevalence of secondary surgeries during the national conference [13], we achieved a comparable VA outcome and retinal attachment rate in the COVID-19 pandemic group compared with the pre-COVID-19 group, which may suggest that adjustment on the treatment pattern according to patient characteristics and RRD service is effective in treating RRD patients during the COVID-19 Pandemic.

### Limitations

Our study’s principal limitation was that this was a retrospective study in a tertiary eye center. In pre-COVID-19, there was a high percentage of complicated patients from cities other than Beijing for consultation at our center. Conversely, in the COVID-19 Pandemic, more local patients came to our center due to Beijing’s lockdown. Patient selection bias is unavoidable. A delay in the treatment can produce a worse clinical situation due to the progression of PVR, CD [50], which are known as the most common cause of failure in retinal detachment surgery [49, 51]. We failed to show the effect of presurgery waiting time and severity of PVR on the outcome of surgery or the choice of surgery. The patients selection bias between the two groups may take account for it. The study was carried out in Beijing Tongren Eye Center, where the retinal specialists were trained on PPV and SB. It cannot reflect China’s procedure choice during the COVID-19 Pandemic, but can reflect Beijing’s condition as the Beijing Tongren eye center was the only center in Beijing that provides RRD service during the COVID-19 Pandemic. Since the most of surgeons thought the follow-up for the IOP control in steroid-related glaucoma was difficult in the COVID-19 Pandemic, there may be treatment bias related to use of intravitreal steroid between the two groups [49, 51].

## Conclusions

The treatment patterns were mainly based on the patients’ presurgery characteristics. The COVID-19 Pandemic impacts the patients’ presurgery characteristics and surgeons’ considerations, which can lead to increased PPV and decreased SB in the COVID-19 Pandemic. The adjustments on the treatment lead to comparable surgery outcomes.

## Data Availability

The dataset(s) supporting the conclusions of this article is (are) available in zhao, meng (2021), “Impact of Covid-19 on RRD”, Mendeley Data, V1, doi: 10.17632/dwx3w4mct2.1.

## Abbreviations

AIC: Akaike information criterion
CD: Choroidal detachment
COVID-19: Coronavirus disease
BCVA: Visual acuity
IOP: Intraocular pressure
IOL: Intraocular lens
MH: Macular hole
PM: Pathological myopia
PVD: Posterior vitreous detachment
PVR: Proliferative vitreoretinopathy
ROC curve: Receiver operating characteristic curve
RRD: Rhegmatogenous retinal detachment
RD: Retinal detachment
SD: Standard deviation
